# Exogenous enzymes unlock prebiotic potential of *Ulva lactuca*: Boosting gut health and growth performance in broilers

**DOI:** 10.1016/j.psj.2025.105834

**Published:** 2025-09-11

**Authors:** Anas Abdelqader, Zeinab M.H. Mahasneh, Veerle Van Hoeck, Mohannad Abuajamieh, Mohamed Abedal-Majed, Mohmmad Al-Qaisi, Rabie Irshaid, Ja'far Al-Khaza’leh

**Affiliations:** a Department of Animal Production, School of Agriculture, The University of Jordan, Amman 11942, Jordan; b Department of Animal Production and Protection, Faculty of Agriculture, Jerash University, Jerash 26150, Jordan; c Kemin Europa N.V., Animal Nutrition and Health, 2200 Herentals, Belgium; d Department of Nutrition and Food Processing, Faculty of Agricultural Technology, Al-Balqa Applied University, P.O. Box 19117, Al-Salt, Jordan

**Keywords:** Broiler chicken, Digestibility, Microbiota, Seaweed, *Ulva Lactuca*

## Abstract

The present study investigated the effects of supplementing broiler chicken diets with green seaweed *Ulva lactuca* (**SW**), an enzyme mixture (**Enz**), or their combination (**SW+Enz**) on growth performance, immune index, intestinal morphology, nutrients digestibility, cecal microbiota balance, and cecal short chain fatty acid (**SCFA**) concentrations. A total of 960 one-day-old male Ross 308 broiler chickens were reared under standard management practices for 35 days. Four dietary treatments were randomly assigned across 12 replicates per treatment (20 chicks each). The treatments included: (C) a control diet (basal); (Enz) control diet supplemented with Enz (250 mg/kg); (SW) a SW diet (10 g/kg); and (SW+Enz) SW+ Enz combination (at the aforementioned doses). Results showed that all supplemented groups significantly improved body weight gain and feed conversion ratio (**FCR**) (*P* < 0.05) compared to the control, with the SW+Enz group achieving the highest values. Specifically, FCR improved by 7.4 % (*P* < 0.05) with individual SW or Enz supplementation, and by 12.3 % (*P* < 0.05) with the SW+Enz combination, all relative to the control diet. Supplementation with either Enz or SW alone enhanced (*P* < 0.05) the digestibility coefficients for crude protein and gross energy (**GE**). Additionally, these individual treatments led to an increased retention (*P* < 0.05) of AME and AMEn when compared to the control group. The combination of **SW+Enz** led to significantly greater (*P* < 0.05) improvements in the digestibility coefficients of crude fat, starch, GE, NDF, ADF, AME, and AMEn when compared with individual inclusion. Relative to the control, single SW inclusion significantly enhanced energy retention by 4 % and protein retention by 6 % (*P* < 0.05). The synergistic effect of combined SW+ENZ further improved energy retention by 9.1 % and protein retention by 11.7 % (*P* < 0.05). The SW+Enz combination demonstrated significant improvements (*P* < 0.05) across several parameters, including the immune index, intestinal villus height, and villus surface area, surpassing other treatment groups. Furthermore, this combination resulted in higher counts of beneficial cecal bacteria and increased SCFA concentrations (*P* < 0.05) compared to supplementing with SW alone. These findings highlight SW+Enz as a promising sustainable feed additive for poultry production, aligning with growing interest in alternatives that enhance productivity while reducing reliance on antibiotics and synthetic growth promoters.

## Introduction

The poultry industry confronts escalating pressure to identify sustainable alternatives to antibiotic growth promoters (AGPs), primarily due to global restrictions enacted over antimicrobial resistance concerns (WHO, 2017). Despite their historical utility in managing intestinal inflammation, mounting apprehensions regarding antibiotic resistance and potential residual effects on human health have necessitated widespread prohibitions; this trend is anticipated to proliferate globally. As a result, safer alternatives like probiotics and prebiotics have gained attention for their ability to improve gut health and regulate immune function in poultry (Abdelqader and Al-Fataftah, 2016; Abdelqader et al., 2020; Abuajamieh et al., 2025). Among these, prebiotics are particularly promising as they enhance the symbiotic relationship between the host and gut microbiota, ultimately improving host health (Sarangi et al., 2016). Unlike probiotics, prebiotics offer the additional advantage of easier large-scale production. The definition of prebiotics has been refined by experts as "a selectively metabolized substrate that confers a health benefit through host-microbial enzymatic activity" (Gibson et al., 2017). Seaweeds, which are natural sources of marine macroalgae, are especially noteworthy. Their diverse bioactive profile includes demonstrated prebiotic, antioxidant, antimicrobial, and anti-inflammatory properties (Makkar et al., 2016; Gullón et al., 2020). Seaweeds, particularly *Ulva lactuca*, have emerging as promising candidates for dietary supplementation due to their rich content of polysaccharides, distinctive amino acid profile (Wong and Cheung, 2001; Gullón et al., 2020) and bioactive compounds that modulate gut health (Makkar et al., 2016; Gullón et al., 2020). Previous studies have emphasized their potential to enhance fiber digestibility and strengthen disease resistance (Wassie et al., 2021; Kulshreshtha et al., 2020; Paul et al., 2021) and has already shown dietary application in poultry nutrition (Abudabos et al., 2013). Its sulfated polysaccharides exhibit immunomodulatory and prebiotic properties across various species (Evans and Critchley, 2014; de Jesus Raposo et al., 2016; Cherry et al., 2019; Øverland et al., 2019; Jasem et al., 2024). The gut plays a crucial role in supporting the rapid growth potential of broiler chickens. Nevertheless, a significant limitation of incorporating seaweed into poultry diets is its high content of non-starch polysaccharides (**NSPs**). These NSPs can act as anti-nutritional factors, consequently impairing nutrient utilization (Tabarsa et al., 2018; Cherry et al., 2019; Gullón et al., 2020). Moreover, conflicting evidence from mammalian models suggests limited fermentability of *Ulva*-derived polysaccharides by gut microbiota (Andrieux et al., 1998; O'Sullivan et al., 2010; Jiao et al., 2011), underscoring the critical need for species-specific validation in poultry.

Enzymatic hydrolysis offers a targeted solution to the challenges posed by high **NSPs** in seaweed. Recent advances in enzymatic pretreatment methods have shown promising results in overcoming this issue (Peñalver et al., 2020). *In vitro* studies display that enzymatic hydrolysis effectively breaks down complex seaweed cell wall matrices, releasing entrapped nutrients and generating bioactive oligosaccharides (Rodrigues et al., 2016; Charoensiddhi et al., 2017; Yaich et al., 2017). This enzymatic approach has also proven effective *in vivo* for other NSPs-rich feed ingredients. For example, Van Hoeck et al. (2021) provided mechanistic evidence that xylanase pretreatment of wheat bran effectively depolymerized structural polysaccharides, as visualized by scanning electron microscopy. Their work further established that this enzymatic hydrolysis generated bioactive oligosaccharides with prebiotic activity while enhancing nutrient bioavailability through cell wall matrix degradation in laying hens. This mechanistic insight highlights the potential of targeted enzymatic processing to significantly improve both the health benefits and nutritional value of NSPs-rich feed ingredients like seaweed.

Despite the established benefits of exogenous enzymes in improving NSP digestibility and the demonstrated prebiotic effects of *Ulva lactuca* (Evans and Critchley, 2014; Øverland et al., 2019), research exploring their combined, synergistic impact in poultry is notably absent. This represents a critical knowledge gap impeding the practical application of these feed additives. Despite the established benefits of exogenous enzymes in improving NSP digestibility and the demonstrated prebiotic effects of *Ulva lactuca* (Evans and Critchley, 2014; Øverland et al., 2019), research exploring their combined, synergistic impact in poultry is notably absent. This represents a critical knowledge gap impeding the practical application of these feed additives. Consequently, this study was designed to investigate the novel synergistic effects of *U. lactuca* and a commercial enzyme mixture. This research is intended to provide foundational evidence for optimizing seaweed utilization in poultry nutrition, concurrently mitigating the limitations associated with NSPs and other anti-nutritional factors. We hypothesize that enzymatic hydrolysis of *U. lactuca* will enhance its prebiotic activity. To test this, the study evaluates the effects of dietary *U. lactuca* (SW), a multi-enzyme blend (Enz), and their combination (SW+Enz) on broiler performance, nutrient utilization, gut morphology, cecal microbiota, SCFA profiles, and immune response, providing the first evidence for optimizing seaweed use in poultry diets through enzymatic pretreatment.

## Materials and methods

### Seaweed (SW) collection and analyses

The green SW (*Ulva lactuca*) was collected from the Red Sea shore, Aqaba-Jordan, then washed with fresh water to remove salts, dried in shade, and then in oven overnight at 60 °C. The SW was ground to pass through a 2 mm sieve using a mill grinder (Retsch ZM 100, Retsch GmbH and Co., K.G., Haan, Germany). Representative SW samples were analyzed according to the methods of the Association of Official Analytical Chemists for dry matter (**DM**), gross energy (**GE**), crude protein (**CP**=N × 6.25), crude fiber, crude fat (ether extract), neutral detergent fiber (**NDF**), acid detergent fiber (**ADF**), crude fiber, and ash. Non-starch polysaccharides (NSP) were determined using the Englyst method (Englyst et al., 1994). Nutrients content of *Ulva lactuca* are presented in Table 1.

**Table 1:** Chemical composition of the *Ulva lactuca*.

Analyzed composition	% DM
DM	88.82
Gross energy (kcal/kg)	2655
Crude protein	21.4
Crude fat	0.41
Crude fiber	13.72
NDF	25.5
ADF	12.1
NSP	35.41
Ash	24.2

DM, Dry matter; NDF, Neutral Detergent Fiber; ADF; Acid Detergent Fiber, ADF; Non-starch polysaccharides, NSP.

### Birds housing and experimental design

All procedures strictly adhered to institutional ethical guidelines and national regulations governing animal research. The animals used in this study were reared and treated in compliance with the Directive 2010/63/EU covering the protection of the animals used for experimental or other scientific purposes.

The experiment was carried out at the University of Jordan’s Poultry Research Farm. One-day-old male broiler chicks (Ross 308) with uniform genetics and initial body weight (46 g ± 0.04) were sourced from a commercial hatchery. Birds were reared on wood shavings with ad libitum access to feed and water, following Ross 308 management and nutritional guidelines. An environmentally controlled poultry house was used for this trial. Forty-eight identical floor pens (1.6 × 1.25 m) were installed in the house and each pen was considered as a replicate. Wood shavings were used as a litter. Lightening protocol and air temperatures were provided according to the Ross 308 strain guidelines.

For this 35-day trial, a completely randomized design was employed, allocating chickens to one of four experimental diets. The first group served as the control (C), receiving a basal diet. The remaining groups were fed basal diets supplemented as follows: with enzymes at 250 mg/kg (Enz group), with dried *Ulva lactuca* at 10 g/kg (SW group), or with a combination of dried seaweed (10 g/kg) and enzymes (250 mg/kg) (SW+Enz group). The experiment included 12 pens per treatment group, housing 20 chicks per pen, totaling 240 birds per treatment group and 960 birds overall. Pens were randomly assigned to treatments, and initial body weights were balanced across all groups to minimize bias.

The experimental diets were formulated to be iso-caloric and iso-nitrogenous, meeting or exceeding the nutrient requirements for broilers as per NRC (1994). The feeding program was divided into three phases: starter (Days 1-14), grower (Days 15-28), and finisher (Days 29-35).

Dietary treatments involved *Ulva lactuca* supplementation at 10 g/kg of feed, which equivalently replaced corn. NSP-degrading enzymes were top-dressed at 250 mg/kg of feed. Both supplements were introduced from Day 1 through Day 35. The enzyme dosage adhered to manufacturer specifications, while the *Ulva lactuca* inclusion rate was based on the study by Abudabos et al. (2013). Complete diet formulations and their nutritional profiles are detailed in Table 2.

**Table 2:** Ingredients and nutrient composition of experimental diets.

Ingredient, %	Starter diets D0–D14	Grower diets D15–D28	Finisher diets D29–D35
Corn (7.3 %)	44.16	49.28	53.57
SBM (46.3 %)	38.24	33.34	29.61
Wheat (11.6 %)	10.00	10.00	10.00
Soybean Oil	3.23	3.46	3.45
MCP	1.57	1.34	1.05
Limestone	1.44	1.30	1.18
Methionine	0.34	0.32	0.28
Salt	0.27	0.27	0.27
Lysine	0.22	0.21	0.19
Na bicarbonate	0.15	0.11	0.10
Threonine	0.10	0.10	0.10
Valine	0.08	0.07	0.05
Premix Vit & Min^1^	0.20	0.20	0.15
Total	100.0	100.0	100.0
**Analysed Nutrients Composition (%)**			
Crude protein %	22.58	20.65	19.15
Crude fat %	5.15	5.46	5.52
Crude fibre %	2.54	2.48	2.45
Starch %	36.71	39.80	42.42
NDF %	7.03	6.96	6.93
ADF %	3.87	3.73	3.64
NSP %	10.92	10.70	10.54
Calculated Nutrients Composition (%)*			
AME kcal/kg	2925.0	3000.0	3050.0
Calcium %	0.90	0.80	0.70
Dig. Phosphorus %	0.42	0.37	0.31
Dig. Lysine %	1.22	1.10	1.00
Dig. Met %	0.61	0.57	0.51
Dig. Met+Cys %	0.90	0.84	0.76
Dig. Threonine, %	0.79	0.73	0.66
Dig. Tryptophan, %	0.25	0.22	0.20

1 Composition of Vitamin-Mineral Premix (per kg): Vitamin A: 12,500,000.00 IU; Vitamin D3: 5,000,000.00 IU; Vitamin E: 70,000.00 mg; Vitamin K3: 3,800.00 mg; Vitamin B1: 2,500.00 mg; Vitamin B2: 7,500.00 mg; Vitamin B6: 4,300.00 mg; Vitamin B12: 25,000.00 mcg; Pantothenic acid: 13,000.00 mg; Niacin (B3): 50,000.00 mg; Folic Acid: 1,000.00 mg; Biotin: 200,000.00 mg; Mn (Oxide): 62,000.00 mcg; Fe (Sulphate): 44,000.00 mg; Zn (Oxide): 50,000.00 mg; Cu (Sulphate): 10,000.00 mg; I (K-iodide): 1,300.00 mg; Se (Na-selenite): 230.00 mg. * Calculated based on NRC (1994).

The NSP enzyme blend used in this study was a commercial multi-enzyme complex (KEMZYME® Plus dry, Kemin Industries), containing three carbohydrases (xylanase, β-glucanase, and cellulase) and two endogenous-like enzymes (amylase and protease). The enzymes have the following minimum activity: (1) Endo-1,3(4)-beta-glucanase: 2350 U/g (588 U/kg feed), (2) Endo-1,4-beta-glucanase: 18000 U/g (cellulase) (4500 U/kg feed), (3) Alpha-amylase: 400 U/g (100 U/kg feed), (4) Bacillolysin: 1700 U/g (protease) (425 U/kg feed), and (5) Endo-1,4-beta-xylanase: 35000 U/g (8750 U/kg feed).

### Performance parameters

Body weight and feed intake were determined per pen (replicate) on weekly intervals using a digital balance. Feed intake was determined by subtracting residual feed from the amount offered at the end of each week. Average daily gain and average daily feed intake were calculated for D 1–14, D15–35 and for the entire experimental period (D 1–35). Feed conversion ratio (FCR) was calculated as grams of feed consumed per gram of live body weight gain. Throughout the trial, we monitored the general health of the flock daily, recording any mortality. The FCR was then corrected for mortality to ensure accurate performance assessment.

### Digestibility Trial

#### Trial settings

On day 26, 48 birds (1 per replicate) were selected based on proximity to the flock’s average body weight (±5 % deviation threshold) to minimize weight variability. Selected birds were individually housed in wire-mesh floored cages equipped with fecal collection trays, maintained in a climate-controlled room (20–22 °C) with 20 h light:4 h dark photoperiod. Feed and water were provided *ad libitum*. Individual body weight and feed intake were recorded daily. Nutrient digestibility was assessed following the EU-reference total excreta collection method (Bourdillon et al., 1990), with a 5-day adaptation period prior to 4-day excreta collection. Titanium dioxide (TiO_2_) was incorporated into the same experimental diets above at a concentration level of 5 g/kg as an indigestible marker. TiO_2_ was included in the diets during the adaptation and the collection periods. Fresh excreta were collected twice daily from plastic trays beneath each cage, with careful removal of contaminants (feathers, scales). Samples from individual birds were pooled daily, homogenized (commercial mixer), then freeze-dried and ground (0.5 mm sieve; Retsch ZM 100 mill, Germany). Processed samples were stored at 4 °C for later analysis. Feed intake and excreta output were recorded per bird throughout the 4-day collection period. Dry matter, nitrogen (Dumas combustion), and gross energy (bomb calorimetry) were analyzed in duplicate. Apparent metabolizable energy corrected for nitrogen (AMEn) was calculated for the whole excreta collection period (D31-D34) using the EU-reference formula (Bourdillon et al., 1990), accounting for individual bird intake and excretion data.

#### Chemical analysis

Diets, SW, and excreta samples were analyzed according to the methods of the Association of Official Analytical Chemists for dry matter (DM: method 934.01), ash (method 923.03), nitrogen (N: method 988.05), crude fiber (method 962.09), and crude fat (method 920,39C). A bomb calorimeter (IKA®–WERKE, C5001, Germany) was used to assess the gross energy (GE). Crude protein (CP) was determined by Nitrogen analyzer (NA 2000, Fisons Instruments, Milano, Italy). Titanium dioxide was quantified using UV absorption spectrophotometry (UVIKON 810, TEGIMENTA, Switzerland) following Short et al. (1996) and Myers et al. (2004). Following Van Soest et al. (1991), neutral detergent fiber (NDF) and acid detergent fiber (ADF) were analyzed using an Ankom-200 system (Ankom Technology, NY). Starch was determined using a commercial assay kit (Megazyme, Boronia, Victoria, Australia) using heat-stable α amylase and amyloglucosidase (McCleary et al., 1997).

#### Calculations

The apparent total tract digestibility (ATTD) of nutrients according to the following formula (Cerrate et al., 2019):

$$\text{ATTD}\;\left(\%\right)=\frac{\left[\text{Nutrient}diet-\left(\frac{\text{TiO}2diet}{\text{TiO}2excreta}\;\times \;\text{Nutrient}excreta\right)\right]}{\left[\text{Nutrient}diet\right]}\;\times \;100$$

The apparent metabolizable energy (AME) was calculated according to the following formula (Cerrate et al., 2019):

$$\text{AME}\;\left(\text{kcal}/\text{kg}\;\text{diet}\right)=\;\text{GE}diet\;-\left(\text{GE}excreta\;\times \frac{\text{TiO}2diet}{\text{TiO}2excreta}\right)$$

The apparent metabolizable energy corrected for nitrogen (AMEn) was calculated by correction for zero nitrogen (N) retention by assuming 8.22 kcal/g nitrogen retained in the body according to the following formula (Hill and Anderson, 1958):

AMEn(kcal/kgdiet)=AME−(8.22×NR)

The nitrogen retention (NR) per gram of diet was calculated according to Hill and Anderson (1958):

$$\text{NR}=\;Ndiet\;-\left(Nexcreta\;\times \frac{\text{TiO}2diet}{\text{TiO}2excreta}\right)$$

The NR values were adjusted by subtracting uric acid nitrogen, quantified following Marquardt (1983), from total excreta nitrogen

#### Intestinal morphology parameters

At the end of the trial (35 day of age), one bird was randomly selected from each pen (replication). A total of 48 birds were selected. After euthanasia, the birds were eviscerated, and the small intestines were carefully extracted. A 2-cm section was collected from the duodenum's mid-region, rinsed in physiological saline, and fixed in 10 % buffered formalin for 24 hours. Tissue samples were dehydrated in graded ethanol solutions, cleared in xylene, and paraffin-embedded. Sections (5 µm thick, three per sample) were microtome-cut, mounted on slides, and H&E-stained. Villus morphology (height, basal/apical width, surface area, and crypt depth) was analyzed using a Leica image analyzer (Leica Microsystems, UK) as described by Abdelqader et al. (2020). Absorptive epithelial cell area measurements followed Yamauchi et al. (2006).

#### Microbiological analysis of cecal contents

Immediately following euthanasia, cecal contents were aseptically collected into sterile bags. Each sample was divided into two aliquots: one for immediate microbial enumeration and another stored at –30 °C for subsequent SCFA analysis. For bacterial quantification, we followed the standardized protocols of Hu et al. (2012) with modifications (Abdelqader et al., 2013). Cecal contents were diluted 1:10 (w/v) in sterile physiological saline (0.9 % NaCl) and homogenized for 3 minutes using a stomacher. Serial decimal dilutions (10⁻¹ to 10⁻⁷) were prepared, and 100 µL aliquots were plated in duplicate on selective media. Duplicate 0.1 mL aliquots of each diluted sample were plated on selective media for microbial enumeration: Rogosa agar (*Lactobacillus* spp.), Beerens agar (*Bifidobacterium* spp.), Reinforced Clostridial agar (*Clostridium* spp.), and MacConkey agar (*Coliforms*). Anaerobic incubation at 37 °C for 48 h was used for *Lactobacillus, Bifidobacterium*, and *Clostridium*, while *Coliforms* were incubated aerobically at 37 °C for 24 h. Colony counts were normalized to fresh digesta weight and expressed as log₁₀ CFU/g. All procedures were conducted under laminar flow to maintain sterility.

#### Cecal Short chain fatty acids (SCFA) content

Cecal SCFA concentrations were quantified via gas chromatography following Zhao et al. (2006) and Kulshreshtha et al. (2014). After thawing, 1 g of cecal digesta samples were treated with 0.2 mL of 24 % metaphosphoric acid, diluted with 4 mL deionized water, and centrifuged (25,000 × g, 20 min, 4 °C). Supernatants were analyzed for acetic, propionic, and butyric acids using an HP 5890 series II gas chromatograph coupled with an HP 5973 mass spectrometer (Palo Alto, CA).

#### Immune index

On day 35, twelve birds from each treatment group were randomly selected for blood sampling. Blood (4 ml) was drained from the jugular vein of each bird using plain tubes. Samples were centrifuged at 115 × g for 15 min to obtain the serum. Commercial ELISA kits specific for chicken were provided by MyBioSource (MyBioSource, Inc. San Diego, CA, USA) and used to analyze the serum levels of immunoglobulin Y (IgY Cat. No. MBS760369), immunoglobulin M (IgM Cat. No. MBS706158), and immunoglobulin A (IgA Cat. No. MBS564152). The analyses were conducted following the instructions provided by the manufacturer of the kits. The above birds were weighed live, euthanized, and dissected to collect the bursa of Fabricius, thymus, and spleen. Absolute organ weights were measured and normalized to body weight to calculate relative organ indices using the formula: Organ Index ( %)=(organ weight/body weight) × 100

### Statistical analysis

Data were analyzed using the GLM procedure in SAS software (Version 9.4). The model included the effects of enzymes (Treated vs. Untreated), seaweed (With vs. Without), replicates, and their interactions. Each replicate was considered as the experimental unit. Differences were accepted as representing statistically significant differences when *P* < 0.05. Tukey’s test was used to separate means for significant interactions.

## Results

### Effect of SW and enzymes on performance parameters

The effects of dietary *Ulva lactuca* (SW), an enzyme mixture (Enz), and their combination (SW+Enz) on broiler growth performance are summarized in Table 3. During the starter phase (1-14 days), broilers fed diets supplemented with SW, Enz, or SW+Enz exhibited significantly higher average daily gain compared to the control group (*P* < 0.05). Specifically, ADG was improved by 5.5 %, 7.9 %, and 7.9 % for Enz, SW, and SW+Enz, respectively, compared to the control (Table 3). From 15 to 35 days, the SW+Enz group maintained the highest ADG, which was significantly greater than the C and SW groups (*P* < 0.05). Overall (1-35 days), broilers in the supplemented groups showed a significantly higher ADG (*P* < 0.05) in comparison to the control group.

**Table 3:** Effects of *Ulva lactuca*, enzyme mixture, and their combination on performance of broiler chickens.

Treatment	Average daily gain (g)			Average feed intake (g)			Feed conversion ratio (g/g)			Total Mortality %
	Day 1 – 14	Day 15 – 35	Day 1 – 35	Day 1 – 14	Day 15 – 35	Day 1 – 35	Day 1 – 14	Day 15 – 35	Day 1 – 35	
1C	29.2^b^	76.5^c^	57.6^b^	38.3	130.1^a^	93.4^a^	1.31^a^	1.70^a^	1.62^a^	2.08
2Enz	30.8^a^	81.7^b^	61.4^a^	37.73	128.5^ab^	92.2^ab^	1.22^b^	1.57^b^	1.50^b^	1.67
3SW	31.5^a^	80.8^b^	61.1^a^	37.5	127.6^ab^	91.6^ab^	1.19^b^	1.58^b^	1.50^b^	2.50
4SW+Enz	31.5^a^	84.6^a^	63.4^a^	37.0	125.5^b^	90.1^b^	1.17^b^	1.49^c^	1.42^c^	2.08
5SEM	0.32	1.20	0.70	0.41	1.03	0.63	0.018	0.024	0.017	1.30
*P*-values										
Diet	<.0001	0.0045	0.0003	0.0622	0.0126	0.0041	<0.0001	0.0001	<0.0001	0.2522
Enz	0.0098	0.0005	<.0001	0.1834	0.0785	0.0412	0.0037	<0.0001	<0.0001	0.3341
Diet × Enz	0.0140	0.5551	0.2878	0.9678	0.7864	0.7990	0.0521	0.5048	0.2701	0.6254

^a−c^ Means with different superscripts within the same column differ significantly (P<0.05), according to Tukey's test.

1 Control chicks treated with Basel diet.

2 Chicks supplemented with enzyme mixture (Enz; 250 mg/kg).

3 Chicks supplemented with green seaweed *Ulva lactuca* (SW;10 g/kg)

4 Chicks supplemented with (SW+Enz) SW+ Enz combination (at the aforementioned doses).

5 SEM = Pooled standard error of the mean. ^a,b,c^*P*≤0.05

From day 15 to 35, average feed intake was significantly lower in the SW+Enz group at 125.5 g compared to the control group, which was highest at 130.1 g (*P* < 0.05). The synergetic effect of the combined SW+Enz group was obvious on FCR throughout the study (Table 3). The FCR was significantly improved in the Enz, SW and SW+Enz groups throughout the study period (*P* < 0.05). Over the entire experimental period (1-35 days), the Enz, SW, and SW+Enz diets resulted in a significant reduction in FCR compared to the control diet (*P* < 0.05). Total mortality did not differ significantly among the treatment groups (*P* > 0.05)

### Nutrient digestibility and energy utilization

The impact of dietary treatments on apparent total tract digestibility (ATTD) and energy utilization in broiler chickens is presented in Table 4. Broilers fed the Enz or SW+Enz diets showed a significant increase in organic matter digestibility compared to the control group (*P* < 0.05). Crude protein digestibility was significantly higher in the SW+Enz group compared to the control (*P* < 0.05). The Enz or SW groups also showed intermediate CP digestibility, with no significant difference between the SW+Enz and the control groups. The SW+Enz and Enz groups had significantly higher crude fat digestibility compared to the SW and control groups (*P* < 0.05). The digestibility of NDF and ADF as well as apparent metabolizable energy were significantly higher in the SW+Enz group when compared to other treatments (*P* < 0.05). Starch digestibility significantly improved (*P* < 0.05) in both the SW+Enz and Enz groups compared to the control. Furthermore, all treated groups exhibited higher nitrogen retention than the control diet (*P* < 0.05). While the Enz and SW groups had intermediate AME values that were not significantly different from the control, the SW+Enz group significantly increased nitrogen-corrected apparent metabolizable energy (AMEn) compared to all other groups (*P* < 0.05). Crude fiber remained unaffected (*P* > 0.05) by any treatment. Furthermore, no significant enzyme × diet interaction was found for any digestibility parameter.

**Table 4:** Effects of *Ulva lactuca,* enzyme mixture, and their combination on nutrient digestibility and energy utilization of broiler chickens.

Treatment	Organic Matter %	Crude Protein %	Gross Energy %	Crude Fat %	Crude Fiber %	NDF^6^ %	ADF^7^ %	Starch %	Nitrogen Retention g/kg	AME^8^ kcal/kg	AMEn^9^ kcal/kg
1C	74.0^b^	79.5^c^	70.4^c^	77.1^b^	11.6	13.3^b^	5.7^b^	83.2^b^	23.1^b^	3085.0^c^	2894.8^c^
2Enz	77.3^a^	85.8^ab^	74.5^b^	83.2^a^	12.6	15.5^b^	8.6^b^	88.6^ab^	24.9^a^	3267.6^b^	3062.6^b^
3SW	75.7^ab^	84.6^b^	73.2^b^	79.9^ab^	14.3	15.5^b^	8.3^b^	87.0^ab^	24.5^a^	3206.4^b^	3005.2^b^
4SW+Enz	78.0^a^	88.8^a^	76.8^a^	84.6^a^	13.9	20.1^a^	12.7^a^	90.5^a^	25.0^a^	3366.8^a^	3161.5^a^
5SEM	0.62	0.96	0.50	1.43	1.51	1.08	0.87	1.49	0.27	22.0	22.3
*P*-Values											
Diet	0.0651	0.0001	<.0001	0.1398	0.1905	0.0028	0.0004	0.0650	0.0141	<.0001	<.0001
Enz	<.0001	<.0001	<.0001	0.0005	0.8593	0.0031	0.0001	0.0046	0.0001	<.0001	<.0001
Diet × Enz	0.4263	0.2733	0.6341	0.6159	0.6474	0.2634	0.3958	0.5320	0.0212	0.6165	0.7957

^a−c^ Means with different superscripts within the same column differ significantly (P<0.05), according to Tukey's test. ^a,b,c^*P*≤0.05

1 Control chicks treated with Basel diet.

2 Chicks supplemented with enzyme mixture (Enz; 250 mg/kg).

3 Chicks supplemented with green seaweed *Ulva lactuca* (SW;10 g/kg)

4 Chicks supplemented with (SW+Enz) SW+ Enz combination (at the aforementioned doses).

5 SEM = Pooled standard error of the mean.

6 NDF = Neutral detergent fiber

7 ADF = Acid detergent fiber,

8 AME = Apparent metabolizable energy

9 AMEn = Apparent metabolizable energy corrected for nitrogen.

### Intestinal morphology

The effects of dietary treatments on intestinal morphology are summarized in Table 5. Broilers fed the Enz or SW+Enz diets exhibited a significantly increased villus height and crypt depth (*P* < 0.05) compared to the SW and control groups, with the highest values observed in the SW+Enz group. Villus surface area was significantly greater in the SW+Enz and Enz groups (*P* <0 .05) when compared to the SW and control groups. Notably, the combined SW+Enz inclusion improved villus surface area by 14 % (*P* < 0.05) compared to the single inclusion of SW. Additionally, the absorptive epithelial cell area significantly increased in all supplemented groups (*P* <0.05) relative to the control group.

**Table 5:** Effects of *Ulva lactuca,* enzyme mixture, and their combination on intestinal morphology and microbiota of broiler chickens.

Treatment	Villus height, µm	Crypt depth, µm	Villus surface area, mm^2^	Absorptive epithelial cell area, µm^2^	*Lactobacillus,* log_10_ CFU/g	*Bifidobacterium,* log_10_ CFU/g	*Clostridium,* log_10_ CFU/g	*Coliforms,* log_10_ CFU/g
1C	1308.4^d^	133.2^c^	0.357^d^	178.8^c^	4.33^c^	3.66^c^	6.97^a^	6.48^a^
2Enz	1451.0^b^	171.7^a^	0.433^b^	204.4^a^	6.04^b^	4.12^b^	5.13^b^	4.37^b^
3SW	1404.2^c^	166.2^b^	0.401^c^	190.3^b^	5.88^b^	4.11^b^	5.37^b^	4.82^b^
4SW+Enz	1477.1^a^	173.0^a^	0.459^a^	201.1^a^	6.37^a^	5.15^a^	5.22^b^	4.74^b^
5SEM	11.21	6.40	0.24	4.22	0.23	0.18	0.49	0.67
*P*-Values								
Diet	0.0021	0.026	0.0054	0.0114	<0.0001	<0.0001	<0.0001	<0.0001
Enz	0.0011	0.028	0.0027	0.0012	<0.0001	<0.0001	<0.0001	<0.0001
Diet × Enz	0.094	0.121	0.242	0.084	0.068	0.107	0.234	0.114

^a−c^ Means with different superscripts within the same column differ significantly (P<0.05), according to Tukey's test.

1 Control chicks treated with Basel diet.

2 Chicks supplemented with enzyme mixture (Enz; 250 mg/kg).

3 Chicks supplemented with green seaweed *Ulva lactuca* (SW;10 g/kg)

4 Chicks supplemented with (SW+Enz) SW+ Enz combination (at the aforementioned doses).

5 SEM = Pooled standard error of the mean. ^a,b,c^*P*≤0.05

### Intestinal microbial populations

The effects of dietary SW, Enz, or SW+Enz on the intestinal microbiota composition are presented in Table 5. Broilers fed diets supplemented with SW, Enz, or SW+Enz showed significantly greater counts of *Lactobacillus* and *Bifidobacterium* than those on the control diets (*P* < 0.05). The highest counts of these beneficial microbes were observed in the combined SW+Enz group (*P* < 0.05). The addition of SW, Enz, or SW+Enz significantly reduced the intestinal colonization of pathogenic *Clostridium* and Coliforms (*P* < 0.0001).

### Immune index

The effects of the different feed supplements on immunoglobulin concentrations (IgY, IgM, IgA) and immune organ indices (spleen, bursa, thymus) are presented in Table 6. The SW+Enz treatment group exhibited the highest IgY concentration (Table 6), which was significantly greater than the C group (*P* < 0.05). Similarly, IgM levels were significantly elevated in the SW+Enz group compared to all other groups (*P* < 0.05). For IgA, the SW+Enz treatment resulted in the highest concentration (137.9 µg/mL), differing significantly from both the control and Enz groups (*P* < 0.05). However, there was no significant difference between the SW+Enz and SW treatments for IgA (*P* > 0.05). The addition of Enz, SW, or SW+Enz did not significantly affect the spleen index, bursa index, or thymus index compared to the control diet (*P* > 0.05).

**Table 6:** Effects of *Ulva lactuca*, enzyme mixture, and their combination on intestinal morphology and microbiota of immune index broiler chickens.

Treatment	IgY, µg/mL	IgM, µg/mL	IgA, µg/mL	Spleen Index	Bursa Index	Thymus Index
1C	1685.1^b^	437.9^b^	105.3^b^	0.126	0.160	0.339
2Enz	1760.1^ab^	443.3^b^	114.4^b^	0.138	0.165	0.378
3SW	1777.6^ab^	447.5^b^	120.5^ab^	0.149	0.165	0.324
4SW+Enz	1926.4^a^	523.9^a^	137.9^a^	0.150	0.167	0.362
5SEM	52.49	18.29	4.74	0.02	0.01	0.04
*P*-Values						
Diet	0.019	0.019	0.0002	0.321	0.780	0.673
Enz	0.040	0.032	0.008	0.706	0.755	0.317
Diet × Enz	0.487	0.060	0.387	0.741	0.900	0.991

^a−c^ Means with different superscripts within the same column differ significantly (P<0.05), according to Tukey's test.

1 Control chicks treated with Basel diet.

2 Chicks supplemented with enzyme mixture (Enz; 250 mg/kg).

3 Chicks supplemented with green seaweed *Ulva lactuca* (SW;10 g/kg)^.^

4 Chicks supplemented with (SW+Enz) SW+ Enz combination (at the aforementioned doses).

5 SEM = Pooled standard error of the mean. ^a,b,c^*P*≤0.05.

### Effect of SW and enzymes on cecal SCFA

**Fig. 1:**
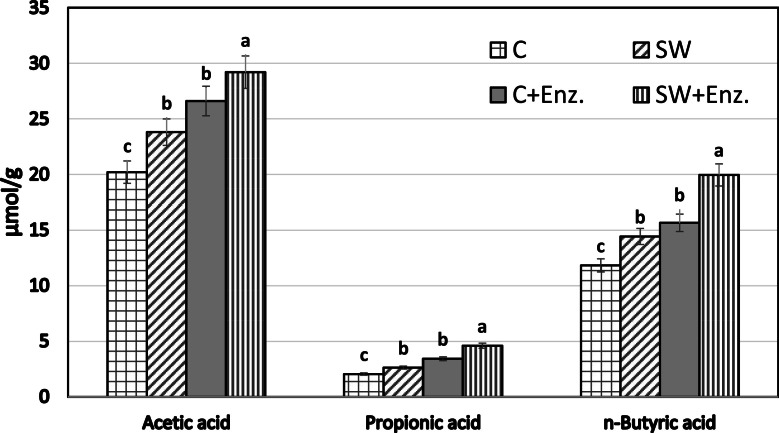
Cecal SCFA levels (μmol per gram digesta) in broiler chickens. Abbreviations are: (C) a control diet (basal); (Enz) control diet supplemented with enzyme mixture (Enz; 250 mg/kg); (SW) a green seaweed *Ulva lactuca* (SW;10 g/kg) and (SW+Enz) SW+ Enz combination (at the aforementioned doses).

The concentrations of short-chain fatty acids (SCFAs; μmol/g of cecal digesta) are presented in Fig. 1. Dietary supplementation with SW and the Enz, either individually or in combination (SW+Enz), significantly increased the concentrations of acetic acid, propionic acid, and n-butyric acid compared to the control (*P* < 0.05). The control group exhibited the lowest production of all measured SCFAs (*P* < 0.05). The highest (*P* < 0.05) SCFA concentrations were observed in the SW+Enz group, indicating a synergistic effect between SW and enzymatic supplementation. While the individual inclusion of SW or Enz alone also enhanced SCFA production compared to the control (*P* < 0.05), their effects remained intermediate and statistically lower than the combined treatment (*P* < 0.05; Fig. 1)

## Discussions

The present study demonstrates that dietary supplementation with *Ulva lactuca* (SW), enzyme mixture (Enz), or their combination (SW+Enz) significantly enhanced broiler performance, nutrient digestibility, gut health, immune response, and cecal SCFA production. Our findings demonstrate that the combined SW+Enz treatment positively influenced multiple aspects of broiler physiology and health more effectively than the individual supplements. The study reveals the synergistic potential of exogenous *Ulva lactuca* enzymes to improve broiler growth and health, ultimately contributing to the sustainability and economic efficiency of poultry production.

The observed superiority in growth indices among chickens fed diets supplemented with SW+Enz relative to control groups suggests a synergistic interaction between these two compounds. This synergistic action is posited to enhance nutrient availability in the SW, consequently improving its digestibility within the intestinal lumen. Consistent with these observed effects, various studies have demonstrated the prebiotic properties of SW (Cherry et al., 2019; Øverland et al., 2019) in improving the growth indices in chicken. Furthermore, Thavasi Alagan et al. (2020) reported an increase in body weight for Aseel chicks whose diets were supplemented with 3 % *U. Lactuca* and 5 % azolla. Conversely, Abudabos et al. (2013) found that the inclusion of 1 % or 3 % SW in broiler diets did not significantly impact growth performance. These effects could be attributed to the NSPs in SW, which impact nutrient digestibility. However, the inclusion of enzymes could enhance nutrient availability and increase digestibility, leading to positive effects on growth attributes in chickens. Enzymes act as catalysts, unlocking *Ulva lactuca*'s latent prebiotic activity (Liu et al., 2025), which demonstrably results in superior gut health (Cañedo-Castro et al., 2019). The beneficial effects of SW and enzyme observed in this study likely stem from several mechanisms. SW contains prebiotic compounds, such as polysaccharides, that can selectively foster the growth of beneficial gut bacteria (Guaragni et al., 2020). The combined action of SW and enzymes may create a synergistic effect: SW provides substrates for microbial fermentation (Cañedo-Castro et al., 2019), while the enzymes enhance the breakdown of complex carbohydrates, ultimately leading to increased SCFA production.

The enhanced average daily gain (ADG) and FCR observed in broilers fed SW, Enz, or the SW+Enz combination strongly suggest these additives improve nutrient utilization and metabolic efficiency. The SW+Enz combination yielded the most significant improvements, particularly during the grower phase (Days 15–35), indicating a powerful synergistic effect. This aligns with prior research demonstrating that enzyme supplementation boosts the digestibility of SW-based diets by breaking down NSPs and other anti-nutritional factors (Costa et al., 2022). The superior performance of birds receiving the combined SW+Enz treatment, compared to individual SW or Enz supplements, further confirms this synergy. This combination notably improved FCR by 12.3 %, directly corresponding to a reduction in feed intake. These findings point to better nutrient absorption and energy utilization, likely due to improved gut function and microbial activity.

As a vital biomarker, nutrient digestibility reflects intestinal integrity and normal digestive function in poultry. The significant improvements in organic matter, protein, fat, and fiber (NDF, ADF) digestibility in the SW+Enz group highlight the role of enzymatic action in unlocking nutrients from *Ulva lactuca*. Seaweeds are rich in complex polysaccharides (e.g., ulvan, cellulose), which are poorly digested by monogastric animals unless supplemented with carbohydrases (Peñalver et al., 2020). The higher nitrogen retention and apparent metabolizable energy (AME) in the SW+Enz group suggest that the combined treatment optimizes protein and energy metabolism. This is particularly relevant for sustainable poultry production, as improved nitrogen utilization reduces environmental nitrogen excretion. The improvement in digestibility coefficients would be an indicator for the improvement in gut health and integrity. As birds eat to satisfy their energy requirements, this indicates that enzymes have extracted extra energy from the diet and make it available for the birds, therefore birds fed enzymes showed a significant decrease in feed intake (Walker et al., 2024). Consistent with our study, Walker et al. (2024) demonstrated that a natural enzyme complex (200 g/ton) in broiler diets led to improvements in digestibility parameters and decreased environmental burden by reducing fecal calcium (Ca) and phosphorus (P) content. This result is further confirmed by the increase in the GE digestibility coefficient and higher AME values compared to the control group. The combined SW and Enz inclusion improved energy retention by 9.1 %, significantly higher than the 4 % observed with single SW supplementation. Furthermore, the SW+Enz combination doubled the value of protein and starch retention compared to individual inclusions. This enhanced energy retention confirms that the enzymes provided additional energy for the birds (Thavasi Alagan et al., 2020). The intestinal morphology results revealed that SW+Enz and Enz groups had significantly taller villi and greater villus surface area, indicating enhanced nutrient absorption capacity. This aligns with studies showing that prebiotic compounds in seaweed (e.g., sulfated polysaccharides) and exogenous enzymes promote enterocyte proliferation and gut barrier function (Kulshreshtha et al., 2020; Walker et al., 2024). A key finding of this study is the positive influence of SW and Enz supplementation on gut microbiota.

Gut microbiotas are critical for nutrient digestibility, directly impacting gut health. Probiotic supplementation in broiler diets can regulate the gut microbiota by decreasing pathogenic bacteria and fostering beneficial bacterial growth. Both individual and combined inclusion of SW and Enz increased populations of beneficial bacteria, specifically *Lactobacillus* and *Bifidobacterium*, while reducing pathogenic colonization by *Clostridium* and *Coliforms*. The most pronounced effects were observed in the SW+Enz group, highlighting its superior impact. A balanced gut microbiota is essential for broiler health, as it enhances digestion, nutrient absorption, immune function, and pathogen resistance (De los et al., 2005).

This shift suggests a prebiotic-like effect, where SW-derived oligosaccharides and enzyme-liberated fermentable substrates selectively promote beneficial bacteria while suppressing harmful ones (Corino et al., 2022). The rise in beneficial microbes aligns with previous studies demonstrating that seaweed polysaccharides stimulate *Bifidobacterium* and *Lactobacillus* growth in humans (Seong et al., 2019) and rats (Hu et al., 2006; Wang et al., 2006). The synergistic effect of SW+Enz further amplified this proliferation, reinforcing the role of prebiotics in modulating gut microbiota to benefit the host. Prebiotics improve gut barrier function, enhance immunity, reduce pathogenic bacteria, and boost short-chain fatty acid (SCFA) production (Cherry et al., 2019; Yaqoob et al., 2021; Tella, 2025). Notably, *Lactobacilli* not only enhance immune responses and cytokine modulation but also inhibit *Salmonella* colonization (Forte et al., 2016; Arreguin-Nava et al., 2019), underscoring their importance in poultry health.

The SW+Enz group exhibited the highest cecal SCFA concentrations (acetate, propionate, and butyrate), indicating enhanced microbial fermentation due to improved polysaccharide breakdown. Butyrate, in particular, serves as a primary energy source for colonocytes and exerts anti-inflammatory effects (Ríos-Covián et al., 2016). The increased SCFA levels in treated groups, especially with SW+Enz, suggest greater fermentation efficiency, consistent with other gut health parameters measured in this study. The SCFAs are crucial for gut homeostasis, lowering intestinal pH to inhibit pathogens while promoting enterocyte regeneration and mucosal integrity (Den Besten et al., 2013). Notably, butyrate enhances intestinal cell structure and repair (Abdelqader & Al-Fataftah, 2016) and has been shown to enhance disease resistance in chickens by inducing antimicrobial host defense peptide gene expression (Sunkara et al., 2011). These effects are amplified when oligosaccharides, released through enzymatic hydrolysis, selectively stimulate beneficial bacteria and SCFA production (Timm et al., 2010). The synergy of SW and enzymes stems from their ability to overcome the "cage effect," where cell wall polysaccharides entrap nutrients like starch, oligosaccharides, and proteins (Khadem et al., 2016; Paloheimo, 2010). The NSP enzymes disrupt this matrix, liberating nutrients for microbial fermentation or host absorption. In *Ulva lactuca*, water-soluble polysaccharides (ulvans) comprise up to 29 % of dry weight, with variable compositions of rhamnose (16.8–45.0 %), xylose (2.1–12.0 %), glucose (0.5–6.4 %), uronic acid (6.5–19.0 %), and sulfate (16.0–23.2 %) (Lahaye & Robic, 2007; Tabarsa et al., 2018; Cherry et al., 2019). Soluble fibers alone account for >45 % of Ulva’s total dietary fiber (Yu-Qing et al., 2016), making it an ideal substrate for enzymatic hydrolysis.

While gut microbiota metabolizes these polysaccharides through their intrinsic enzymatic activity, supplemented exogenous enzymes (e.g., grain-targeted blends) further hydrolyze ulvan into bioactive oligosaccharides (Lahaye & Robic, 2007; Rodrigues et al., 2016). A seaweed-specific enzyme formula would be more efficient, as it targets algal-specific glycosidic linkages (Zaporozhets et al., 2014). Although enzymes like ulvan-lyase (isolated from marine bacteria) show promise (Lahaye et al., 1997), their commercial availability remains limited. Nevertheless, enzymatic hydrolysis effectively disrupts cell walls of marine algae to release prebiotic compounds (Kadam et al., 2013; Wen et al., 2020). The combination of SW and enzymes maximizes prebiotic effects: oligosaccharides released from cell walls are readily fermented by microbiota, elevating SCFA production. These metabolites, along with the oligosaccharides themselves, collectively enhance gut health, nutrient absorption, and pathogen resistance in animals.

The elevated immunoglobulin (IgY, IgM, IgA) levels observed in the SW+Enz group indicate a strengthened humoral immune response. Seaweed polysaccharides are recognized immunomodulators, known to stimulate both gut-associated lymphoid tissue and systemic immunity (Hwang et al., 2022). The absence of significant changes in immune organ indices suggests that the immune stimulation was primarily mucosal rather than systemic. Collectively, these results suggest that *Ulva lactuca* and enzymes can enhance the immune function of broilers, potentially improving their disease resistance.

The findings of this study have important implications for sustainable broiler production. By incorporating *Ulva lactuca* and enzymes into broiler diets, producers can improve animal health and welfare and reduce environmental impact. This study confirms the prebiotic potential of *Ulva lactuca* in broiler diets and demonstrates, for the first time, that enzyme supplementation further enhances its benefits. By leveraging this natural seaweed-enzyme synergy, poultry producers can improve gut health, reduce reliance on synthetic additives like antibiotics, and move toward more sustainable broiler production, without compromising performance

## Conclusions

This study reveals that the combined supplementation of *Ulva lactuca* (SW, 10g/kg) and enzymes (Enz, 250 mg/kg) creates a synergistic effect, significantly boosting broiler growth performance, nutrient digestibility, gut health, and cecal short-chain fatty acid (SCFA) production. The SW+Enz combination consistently outperformed individual treatments, showing superior improvements in feed efficiency, intestinal morphology, and microbiota balance. These benefits likely come from the enzymatic breakdown of SW’s cell wall structure, which frees up fermentable oligosaccharides and intensifies its prebiotic effects. Given its many advantages inhibiting pathogens, modulating the immune system, and promoting gut homeostasis through SCFA production; the SW+Enz strategy offers a promising alternative to traditional antibiotic growth promoters. While these findings align with global demands for sustainable, antibiotic-free poultry production, further on-field validation is needed to confirm its practical applicability.

## CRediT authorship contribution statement

**Anas Abdelqader:** Writing – review & editing, Writing – original draft, Visualization, Validation, Supervision, Software, Resources, Project administration, Methodology, Investigation, Funding acquisition, Formal analysis, Data curation, Conceptualization. **Zeinab M.H. Mahasneh:** Writing – review & editing, Writing – original draft. **Veerle Van Hoeck:** Writing – review & editing, Validation, Resources, Methodology. **Mohannad Abuajamieh:** Writing – original draft, Validation, Methodology. **Mohamed Abedal-Majed:** Writing – review & editing, Validation, Methodology. **Mohmmad Al-Qaisi:** Writing – review & editing, Validation, Methodology. **Rabie Irshaid:** Writing – review & editing, Validation, Methodology. **Ja'far Al-Khaza’leh:** Writing – review & editing, Validation, Methodology.

## Disclosures

The authors declare that they have no conflict of interest in this article.
